# Papillary Renal Cell Carcinomas Demonstrating Micropapillary Features: An Investigation Into the Diagnostic and Prognostic Implications

**DOI:** 10.7759/cureus.24944

**Published:** 2022-05-12

**Authors:** Beatriz Caraballo, Maha Abdulla, Sunder Sham, Guang-Qian Xiao, Pamela Unger

**Affiliations:** 1 Pathology and Laboratory Medicine, Lenox Hill Hospital, New York City, USA; 2 Department of Pathology, Lenox Hill Hospital, Northwell Health, New York City, USA; 3 Department of Pathology, Keck School Of Medicine of University of Southern California, Los Angeles, USA

**Keywords:** micropapillary, kidney, pathology, renal cell carcinoma, urology, papillary

## Abstract

Aims: Papillary renal cell carcinoma (PRCC) with micropapillary carcinoma (MC) has been rarely described. We conducted a retrospective descriptive evaluation of the association of MC with PRCC and the possible prognostic implications.

Methods: A database search was made at the University of Southern California (USC) and Lenox Hill Hospital (LHH; New York City) in June 2016-June 2019 of PRCC cases with MC. Diagnosis of MC was made using routine histology, based on the presence of small clusters of cells without a vascular core. Features evaluated included: percent of MC, gross appearance, PRCC typing, nuclear grade, lymphovascular invasion, and lymph node metastasis.

Results: 848 RCC cases (690 from USC and 157 from LHH); 70 cases PRCC (54 from USC, 16 from LHH) of these cases, 13 had an MC, 12 were from radical nephrectomy, and 12 cases were male. Mean age was 68.3 years; seven were located in the right kidney. Average tumor size was 8.6 cm. MC ranged from 10% to 80% (average 37.5%), nine cases were PRCC type 2 and four type 1. Nuclear grade: three cases (grade 2), nine cases (grade 3), and one case (grade 4); 11 out of 13 tumors presented with extrarenal extension; nine cases that had lymph nodes submitted had metastatic carcinoma.

Conclusions: The presence of a micropapillary component in PRCC was found to be 18.5%, and it was predominantly associated with high pathologic stage and lymph node metastases. The clinical course of these tumors seems similar to MC in other tissues/organ systems. We advocate reporting this pattern when identified.

## Introduction

Papillary renal cell carcinoma (PRCC) is the second most common primary renal neoplasm, with only clear cell carcinoma being more common.

Micropapillary carcinoma (MC) is classically defined as small clusters of neoplastic cells without a vascular core, surrounded by lacunar spaces [1]. It has been suspected that due to their lack of a vascular core, these aggressive cells undergo a particular differentiation to work under hypoxic and low-nutrient conditions. This has been recently described as the up-regulation of receptors glucose transporter 1 (GLUT1) [2] and transcription factor hypoxia-inducible factor (HIF)-1, which controls angiogenesis and accommodates certain cellular activities under hypoxia [3-6].

PRCC possessing a micropapillary component is not well documented in the medical literature. As of this writing, there are few reports available of any RCC with micropapillary features described. First, one case report of a renal mucinous tubular and spindle cell carcinoma [2]. Second, another case report describing micropapillary features in a case of a transcription factor E3 (TFE3) rearrangement RCC [7]. In these two cases, the tumor showed lymphovascular invasion in the area with the micropapillary component, third, and largest study to date studies grading, typing, and architecture of PRCC, and mentions 10 cases with micropapillary architecture showing adverse prognosis in overall survival [8]. The aim of this study is to make a descriptive evaluation of pathologic characteristics in cases of PRCC with a micropapillary architecture component.

## Materials and methods

After institutional review board approval, a retrospective computer search was performed in the University of Southern California (USC) and Lenox Hill Hospital (New York City) databases between June 2016 and June 2019 of patients that had been treated by radical or partial nephrectomy. From this review, cases were selected with the diagnosis of RCC with a final sub-selection of the cases with the diagnosis of PRCC. Stored hematoxylin- and eosin-stained slides were retrieved and reviewed by genitourinary pathologists.

The tumors were classified following the 2016 WHO renal tumor classification. The staging of the patient was done following the 2017 Common Alerting Protocol (CAP) protocols and the 8th Edition American Joint Committee on Cancer (AJCC) Staging Manual. The nuclear grade was determined following the WHO/International Society of Urologic Pathologists (ISUP) grading system [9].

The micropapillary architecture was defined as a nest of cells without fibrovascular cores lying within spaces that do not represent vascular or lymphatic channels. The micropapillary component was given a percentage following evaluation of tumor slides. Only tumors with more than 10% of micropapillary architecture were included in the study. The pathologic parameters evaluated in each tumor were: 1) size in greatest dimension, 2) presence of necrosis and hemorrhage, 3) sinus adipose tissue and extrarenal invasion, 4) lymphovascular invasion, and 5) metastasis in lymph nodes (LN) (when available). All tumors with TFE3 rearrangement confirmed by fluorescence in-situ hybridization (FISH) and succinate dehydrogenase or fumarate hydratase deficiency confirmed by immunohistochemistry were excluded.

## Results

**Figure 1:**
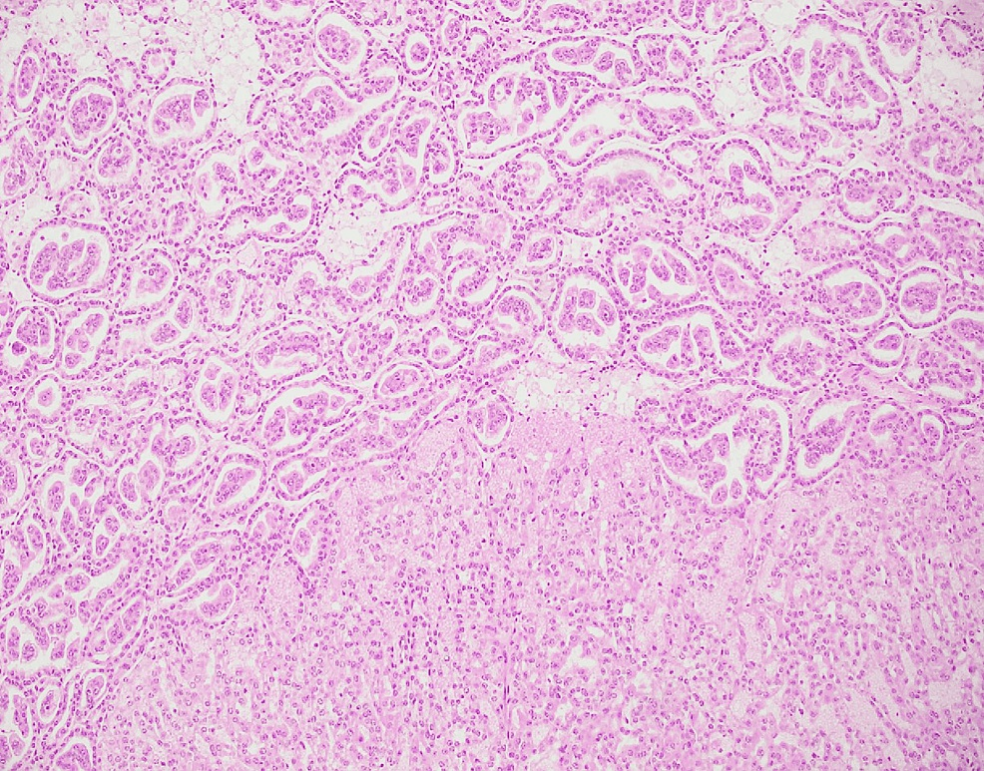
Low-power view of type 1 papillary renal cell carcinoma Type 1 papillary renal cell carcinoma showing cuboidal cells with small round to oval nuclei arranged in a single layer with transition to micropapillary areas (Case 2), Hematoxylin and Eosin (4×).

**Figure 2:**
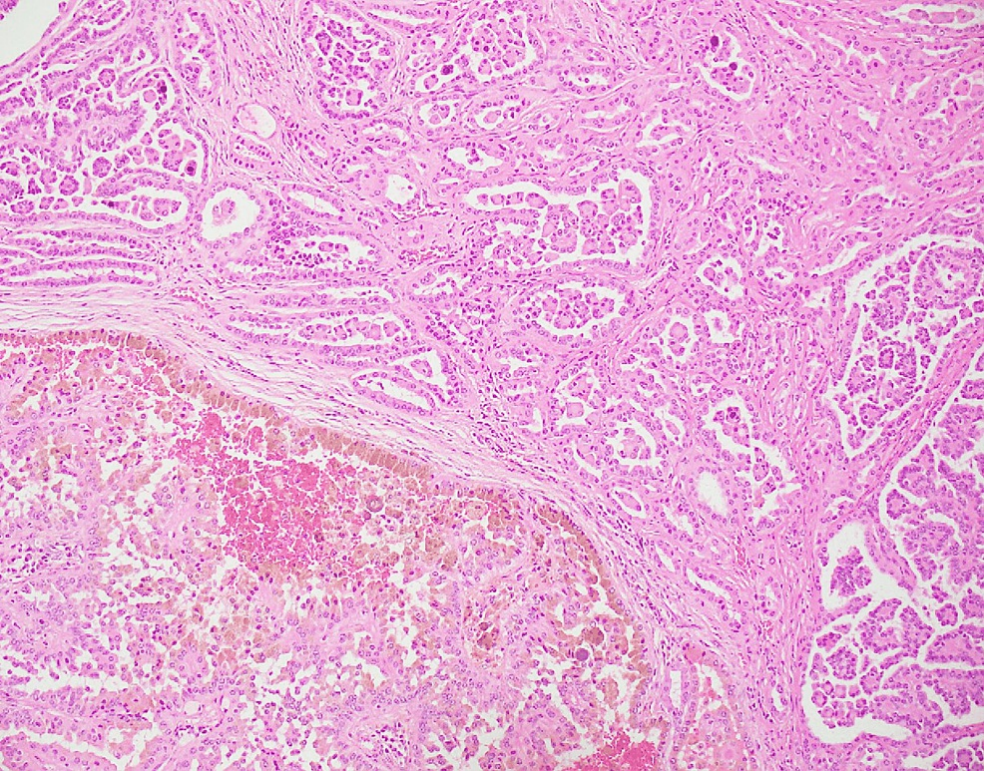
Low-power view of type 2 papillary renal cell carcinoma Type 2 papillary renal cell carcinoma showing pseudostratified layers of cells with eosinophilic cytoplasm, atypical nuclei, and hemorrhage with transition to micropapillary areas (case 3), Hematoxylin and Eosin (4×).

**Figure 3:**
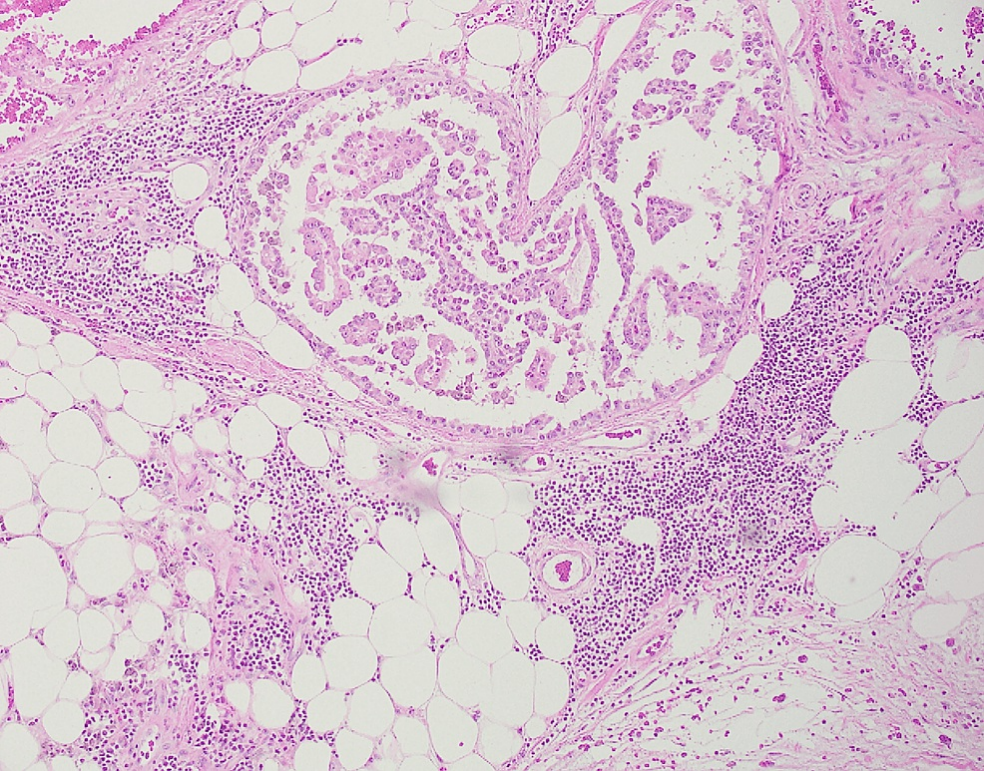
Low-power view of micropapillary carcinoma Micropapillary carcinoma invading fat, tumor cells have eosinophilic cytoplasm, atypical nuclei with prominent nucleoli (Case 11), Hematoxylin and Eosin (4×).

From a total of 848 cases of RCC (partial nephrectomy n = 690 and radical nephrectomy n = 158), we identified 70 cases (8.2%) of PRCC, 54 from USC and 16 from Lenox Hill. Of these 70 cases, a total of 13 cases (18.5%) showed tumors with a micropapillary component (Table 1, Figures 1-2). In cases with micropapillary RCC, patient ages ranged from 43 to 87 years old (mean = 68.3, median = 71). Twelve patients were male (92.3%) and one was female (7.6%). The maximal dimension of the tumor ranged from 2 cm to 16 cm (mean = 8.5 cm, median = 7.2 cm). Six were located on the left (46.1%) and seven on the right (53.8%). Twelve patients underwent radical nephrectomy and one patient underwent partial nephrectomy. Tumor gross appearances varied: two tumors were encapsulated, nine were unencapsulated, and one was partially encapsulated. Seven were poorly circumscribed and six well-circumscribed. Nevertheless, all tumors presented with a variably infiltrative border under the microscope. Two tumors were limited to the kidney (15.3%), seven patients had renal sinus invasion (53.8%) (Figure 3), and three had adrenal gland involvement (23%). In patient number 2, the tumor had direct extension into the right adrenal gland, hepatic parenchyma, and the inferior vena cava, occluding 95% of the lumen. In patients 5, 11, and 13, the tumor invaded beyond the capsule into the major vessels. Seven tumors (53.8%) were positive for necrosis, ranging from 5% to 90% (mean = 50%, median = 50%).

**Table 1:** Micropapillary renal carcinoma tumor characteristics Showing characteristics analyzed per case which include: age, sex, tumor size, laterality, type of surgery, gross appearance, histologic variant, WHO/ISUP nuclear grade, percentage of micropapillary carcinoma, tumor extension, lymphovascular invasion status, lymph node (LN) metastasis status, tumor staging by the 8th Edition American Joint Committee on Cancer Staging (AJCC), and the follow up for each patient. ISUP: International Society of Urologic Pathologists

Case	Age	Sex	Tumor size (cm)	Laterality	Surgery	Gross appearance	Variant	Nuclear grade	Micropapillary component	Tumor extension (microscopic)	Lympho- vascular invasion	LN metastasis	Stage	Follow-up (months)
1	56	M	2	Left	Partial	Poorly circumscribed, tan	Papillary renal cell carcinoma type 1	3	15%	Hilar tissue	Present	Not submitted	pT1 Nx Mx	15 months (no recurrence)
2	55	M	16	Right	Radical	Irregular, tan-yellow, lobulated focal fibrosis and necrosis 50%	Papillary renal cell carcinoma type 1	4	12.50%	Renal sinus, perirenal fat, collecting system, adrenal and liver	Present	Yes	pT4 N1 Mx	6 months (dead after lung metastasis)
3	75	M	7.2	Right	Radical	Well-circumscribed, yellow-tan w/hemorrhage	Papillary renal cell carcinoma type 2	3	35%	Perirenal and sinus fat	Present	Yes	pT3 N1 Mx	42 months, alive with neck metastasis
4	78	M	16	Right	Radical	Irregular, brown w/extensive necrosis 60%	Papillary renal cell carcinoma type 2	3	25%	Renal sinus fat	Present	Yes	pT3 N1 Mx	27 months, lost to follow-up
5	85	F	15.5	Left	Radical	Well-circumscribed, yellow to red, w/hemorrhage and necrosis 90%	Papillary renal cell carcinoma type 2	3	60%	Renal sinus, beyond capsule, into major veins	Present	Yes	pT3c N1 Mx	6 months, lost to follow-up
6	61	M	12.5	Right	Radical	Irregular, tan-white, cystic w/hemorrhage	Papillary renal cell carcinoma type 2	3	50%	Perirenal fat and adrenal gland	Present	Yes	pT4 N1 Mx	7 months, still healthy
7	43	M	4.3	Right	Radical	Irregular, tan-white, solid, w/hemorrhage	Papillary renal cell carcinoma type 2	3	10%	Renal sinus fat (focal)	None	Yes	pT3a N1 Mx	10 months, still healthy
8	71	M	5.5	Left	Radical	Well-circumscribed, gray-tan w/hemorrhage	Papillary renal cell carcinoma type 2	2	80%	Limited to the kidney	None	Not submitted	pT1b Nx Mx	12 months, still healthy
9	87	M	10.2	Left	Radical	Well-circumscribed, yellow-red, w/hemorrhage and necrosis 50%	Papillary renal cell carcinoma, type 1	2	40%	Limited to the kidney	None	Not submitted	pT2b Nx Mx	24 months. Now with two indeterminate pulmonary nodules
10	71	M	6	Right	Radical	Poorly circumscribed, yellow-brown w/necrosis 12.5%	Papillary renal cell carcinoma type 2	3	30%	Perinephric fat	No	Not submitted	pT3a Nx Mx	9 months, still healthy
11	72	M	2 (multifocal)	Left	Radical	Multifocal, poorly circumscribed, yellow-orange, w/hemorrhage	Papillary renal cell carcinoma type 2	3	60%	Perinephric fat, renal sinus, renal and vena cava vein, and adrenal gland	Present	Yes	pT4 N1 Mx	6 months (dead)
12	68	M	2.7	Left	Radical	Well-circumscribed, white-grey, w/necrosis 15%	Papillary renal cell carcinoma type 1	2	30%	Perinephric fat	No	Yes	pT3 N1 Mx	18 months, still healthy
13	67	M	11.7	Right	Radical	Well-circumscribed, encapsulated, tan-brown, w/hemorrhage and necrosis 80%	Papillary renal cell carcinoma type 2	3	40%	Renal vein and perirenal fat	No	Yes	pT3 N1 Mx	7 months, still healthy

**Figure 4:**
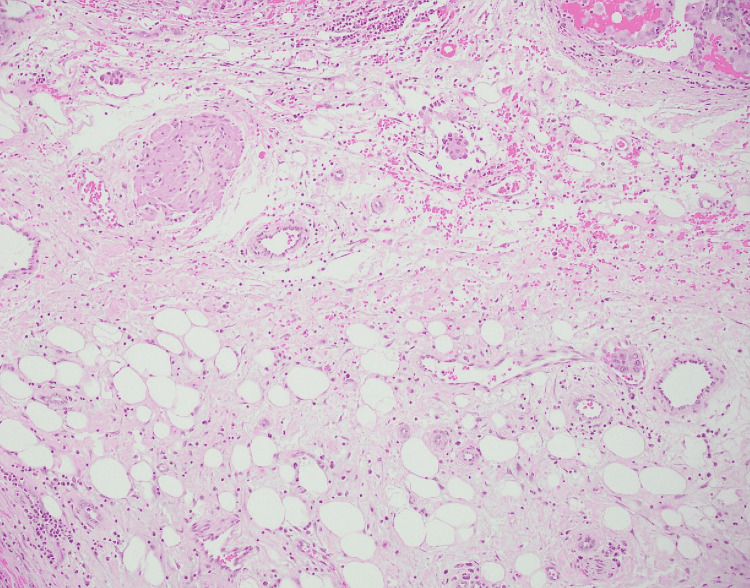
Low-power view of papillary renal cell carcinoma in lymphatics Clusters of papillary renal cell carcinoma with micropapillary features in lymphatics, tumor cells showing eosinophilic cytoplasm and atypical nuclei (Case 2), Hematoxylin and Eosin (4×).

**Figure 5:**
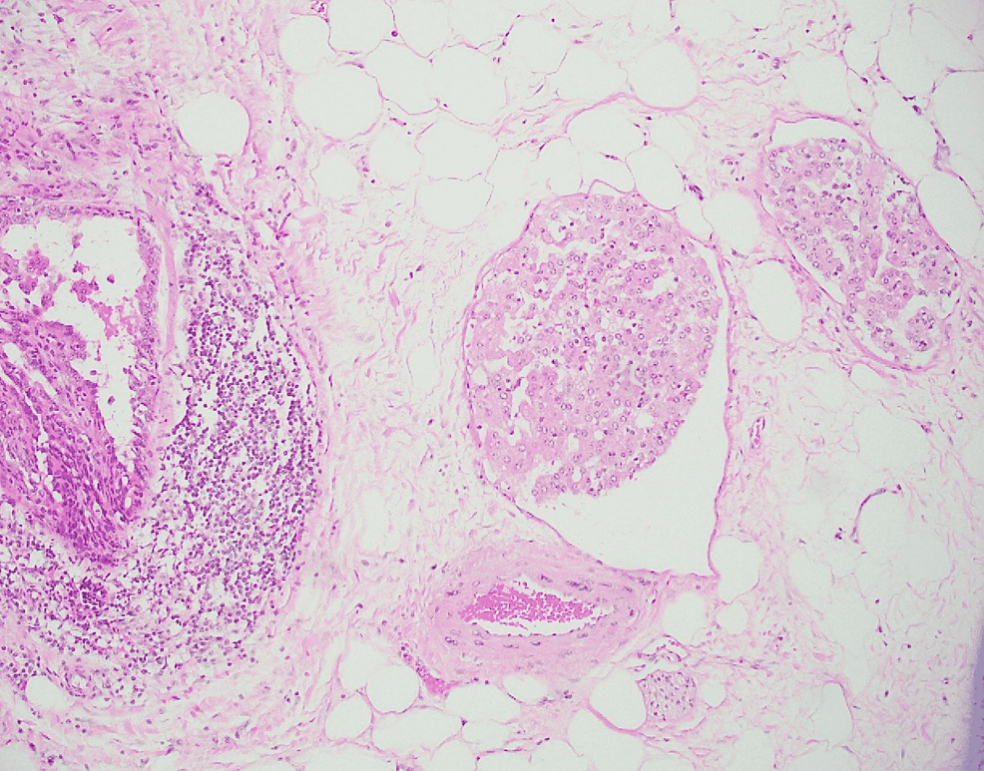
Medium-power view of micropapillary carcinoma with lymphovascular invasion Papillary renal cell carcinoma with micropapillary features with lymphovascular invasion, tumor cells showing eosinophilic cytoplasm with atypical nuclei with prominent nucleoli (Case 4), Hematoxylin and Eosin (10×).

After histologic examination, four tumors (30.7%) were classified as PRCC type 1, and nine tumors (69.2%) as PRCC type 2. On the basis of WHO/ISUP grading criteria: three cases (23%) were grade 2, nine cases (69.2%) were grade 3, and one case (7.6%) was grade 4. Micropapillary architecture was identified in all 13 cases, ranging from 10% to 80% of tumor involvement (mean = 37.5%, median = 35%). Sarcomatoid differentiation was present in one case (patient 2) and involved 50% of the tumor. Lymphovascular invasion of small vessels was present in seven patients (53.8%) (Figures 4-5). Perineural invasion was present in one patient (7.6%).

**Figure 6:**
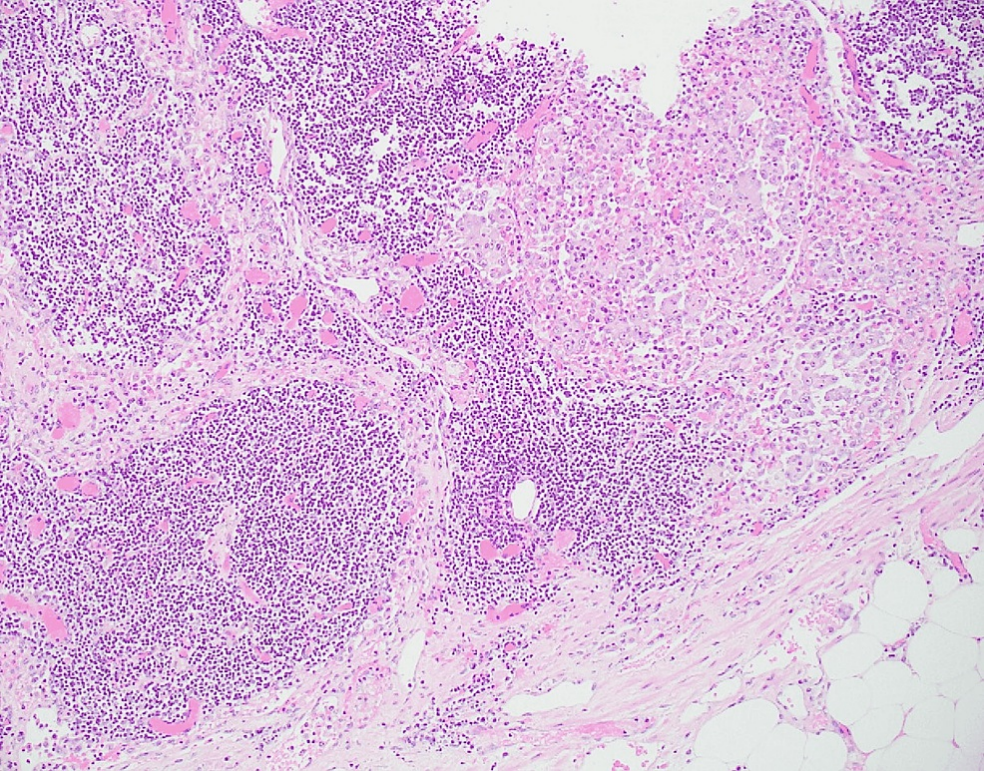
Medium power of metastatic micropapillary carcinoma Lymph node with metastatic carcinoma, tumor cells showing in micropapillary clusters with eosinophilic cytoplasm and atypical nuclei with prominent nucleoli (Case 2), Hematoxylin and Eosin (10×).

Two tumors were stage pT1 (15.3%), one tumor was stage pT2 (7.6%), seven tumors were stage pT3 (53.8%), and three tumors were stage pT4 (23%). LNs were examined in 10 patients (77%). The total number of LNs examined was 113 LN and the number of LNs submitted per patient ranged from 1 to 38 LN (mean = 11.3 LN, median = 6 LN); positive lymph nodes (+LN) per patient ranged from 1 to 38 +LN (mean = 8 +LN, median = 3 +LN) (Figure 6). Locations of the LNs submitted were: para-aortic 40 LN (35.3%), paracaval 34 LN (30%), superior mesenteric 15 LN (13.2%), renal hilar 13 LN (11.5%), retroperitoneal nine LN (7.9%), and one cisterna chyli LN (0.8%). Positive LNs by region varied as follows: paracaval 30/34 +LN, para-aortic 17/40 +LN, superior mesenteric 12/15 +LN, renal hilar 9/13 +LN, retroperitoneal 5/9 +LN, and cisterna chyli 1/1 +LN. More LNs were submitted from patients 3, 4, and 6, where metastatic carcinoma was found in 17/34, 3/13, and 38/38 LNs, respectively. The size of the largest reported metastatic carcinoma ranged from 0.1 cm to 8.6 cm (mean = 2.06 cm, median = 1.05 cm). Patients 2, 4, and 6 had matted LNs, all in the paracaval area, that measured 3 cm, 8.6 cm, and 4.7 cm, respectively. LNs for patients 1, 8, 9, and 10 were not submitted. The mean follow-up was 14.5 months (range: 6-42 months). Of the 13 patients, two (patients 2 and 11) died within 6 months of diagnosis as a result of the extension of the RCC; one patient developed distant neck metastasis 3 years after diagnosis; one patient developed two indeterminate lung nodules one year after diagnosis with no proven biopsy; and two patients were lost in follow-up, patient 4 after 2 years, and patient 6 after 6 months.

## Discussion

MC was initially described by McDivitt et al. in 1982 in breast carcinoma [10]. Subsequently, MC has been described in numerous organ systems including the lung, bladder, and colon with associated frequent lymphovascular invasion, LN metastasis, and higher pathologic stage [2,5,11-15]. The overall aggressivity of micropapillary tumors could be explained because of the observation of the low apoptotic rate of micropapillary tumor cells [12].

PRCC is the second most common renal malignancy. It was described as early as 1976 and included as its own category in the Heidelberg classification in 1977 [16,17]. Kovacs et al. in 1991 found that PRCC was associated with trisomy of chromosome 3q, 7, 8 12, 16, 17, or 20 and in men by loss of the Y chromosome [18].

PRCC is histologically subdivided into type 1 and type 2, but this classification remains controversial [19]. Although there is an immunophenotypic and genetic difference between the two types, there is a high incidence of mixed patterns [8,20-22]. PRCC type 2 is a very heterogeneous group and it has been proposed that genetically different subtypes of PRCC might have different prognostic implications [8,22,23]. Saleeb et al. found that pathways with enrichment of the region 8q and 5p of PRCC type 2 were associated with increased tumor aggressiveness [20]. Clinically, PRCC type 2 was associated with greater stage, grade, and lymphovascular invasion compared with type 1 [24,25]. However, there are analyses that find no significant association between PRCC type 2, disease-free survival, and overall survival [8,21]. In our study series, nine cases of type 2 PRCC had 43.3% micropapillary architecture, while cases with type 1 PRCC had an average of 24.3% of the entire tumor. The majority of studied cases (9/13) had submitted LNs. All nine cases presented positive LNs, and case 6, presented 38 positive LNs. Overall, we have a larger prevalence of micropapillary architecture with PRCC type 2 (n = 10, 76.9%).

One of the most important factors to determine the prognosis of renal malignancies is the pathologic stage [23,26]. Included in the pathologic stage, tumor size is a determinant component of the current classification. In our series, six cases had tumors larger than 10 cm, or as in case 11, the tumor was multifocal. All had variable percentages of micropapillary architecture, making it impossible to correlate a specific percentage of micropapillary architecture with a specific size of the tumor. Tumor grade is another important predictive factor [27]. The grading system has been evolving until the most recent accepted version, the WHO/ISUP grading criteria [28]. In our examined cases, stage 3 and stage 4 cases all had a high ISUP grade. One case that could show the relevance of nuclear grade over micropapillary architecture was case 8, which had the largest micropapillary architecture percentage (80%), but with nuclear grade 2, and the tumor measured 5.5 cm and was stage pT1 (no LN submitted) and the patient has no reported metastasis after 12-month follow-up. However, the size of a tumor partly depends on the duration of tumor growth in the body. Given that the majority of the studied cases presented with LN metastasis regardless of their tumor grade or size, it is consistent that micropapillary pattern can be highly correlated with regional LN (an even distant) metastasis, as seen in other tumors with micropapillary pattern.

With such a small sample, it is not possible to find a strong correlation between the percentage of micropapillary architecture, LN metastasis, and staging. An example of this is the case with the largest percentage (80%) of micropapillary architecture was stage pT1, which might have been related to its relatively small size (5.5 cm), as well as LNs not being submitted for evaluation. In contrast, the case with the lowest percentage (10%) of micropapillary architecture was stage pT3 and had positive LNs. In patient number 2, with 12.5% of micropapillary component, and stage pT4 with LNs metastasis, this can be explained because of the extensive presence of sarcomatoid differentiation. Sarcomatoid differentiation has been previously related to poor prognosis [9,28]. Of the two patients in our series that died within the first 6 months of diagnosis, case number 2 had 12.5% of micropapillary architecture in conjunction with sarcomatoid architecture, and high nuclear grade. In contrast, case 11 had 60% of micropapillary architecture and high nuclear grade.

Yang et al. described micropapillary architecture in 5.4% of their studied cases (10/185), with an involvement ranging from 5% to 30%, and found statistical significance in univariate analysis between micropapillary architecture and worse disease-free survival and overall survival; but they only found statistical significance in multivariate analysis for overall survival [8]. Explaining this phenomenon not just because of the low number of cases but also because they also observed that these features often coexist with other detrimental architecture patterns and/or with high WHO/ISUP grade. We concur with their statement because we also observed micropapillary architecture in association with a high nuclear grade in 10 of our cases. Likewise, we hypothesize that typing of PRCC may not be as important a prognostic factor as adverse architecture. The correlation of micropapillary architecture with lymphovascular invasion and the high potential for metastasis makes the description of micropapillary architecture an important resource for clinicians [1,13]. Our observations indicate that micropapillary architecture could be an important prognostic factor for tumor aggressivity and metastasis. We are also aware that micropapillary features often coexist with high WHO/ISUP grade and that tumor aggressivity is multifactorial. Because of the diversity of genetic alterations in PRCC type 2, more studies are necessary to compare micropapillary architecture, nuclear grade, and genetics.

## Conclusions

This is one of the largest series describing PRCC with micropapillary architecture presenting aggressive behavior. Nevertheless, it is important to mention that our study has two important limitations. First, our cases originated from only two institutions and were therefore limited to the patient population of each institution; second, the number of cases was limited due to the relatively low prevalence of micropapillary architecture in PRCC.

A further larger, multicentric investigation is necessary to determine the threshold value at which micropapillary architecture in renal carcinoma correlates with tumor aggressiveness and worse patient outcomes, providing additional valuable information for better patient management, risk-stratification, and therapy selection.
